# How Does Temperament in Toddlers at Elevated Likelihood for Autism Relate to Symptoms of Autism and ADHD at Three Years of Age?

**DOI:** 10.1007/s10803-021-05001-z

**Published:** 2021-04-14

**Authors:** Linn Andersson Konke, Tommie Forslund, Elisabeth Nilsson-Jobs, Pär Nyström, Terje Falck-Ytter, Karin Brocki

**Affiliations:** 1 Department of Psychology, Uppsala University, Box 1225, 751 42 Uppsala, Sweden; 2 Department of Psychology, Stockholm University, Stockholm, Sweden; 3 Center of Neurodevelopmental Disorders (KIND), Karolinska Institutet, Stockholm, Sweden; 4 Swedish Collegium for Advanced Study (SCAS), Uppsala, Sweden

**Keywords:** Temperament, Autism Spectrum Disorder, Attention-Deficit/Hyperactivity Disorder, Infant Sibling Studies

## Abstract

The current study investigated longitudinal associations between parent-rated temperament, observed exuberance and accelerometer activity level at 18-months and symptoms of ASD and ADHD at 36-months in a sample of 54 children at elevated likelihood for ASD. For the specific parent-rated temperament scales, most observed significant associations appeared to be specific for either ASD or ADHD symptoms. Indeed, by controlling for overlapping symptoms a different pattern of associations emerged. These results illustrate how temperamental measures may signal risk for later ASD versus ADHD symptomatology in infants at elevated likelihood for ASD. In addition, they indicate the potential of adopting a broader view on neurodevelopmental disorders by investigating not only ASD traits, but also co-occurring disorders such as ADHD in samples of elevated likelihood for ASD.

Autism Spectrum Disorder (ASD) is defined by social communication deficits and repetitive and restricted behaviors (Diagnostic and Statistical Manual of mental disorders, DSM-5; American Psychological Association, 2013). Individuals with ASD often have co-occurring conditions, such as attention-deficit/hyperactivity disorder (ADHD), which is characterized by a persistent pattern of inattention and/or hyperactivity/impulsivity (Simonoff et al., 2008). Both ASD and ADHD are childhood-onset neurodevelopmental disorders and share high heritability rates with partially overlapping genetic and etiological factors (Posthuma & Polderman, 2013; Rommelse et al, 2010, 2011a; Rutter et al., 2006).

The early developmental trajectories of ASD and ADHD are far from fully understood. Infant sibling studies, which systematically follow younger siblings to older children with ASD, indicate that certain temperamental characteristics can be spotted already during infancy in relation to later ASD (Johnson et al., 2015). Many studies have linked temperamental differences to ADHD-symptoms (De Pauw & Mervielde, 2010; Nigg, 2006) but only a handful recently published studies have used a familial risk design to understand ADHD symptomatology (Miller et al., 2016a, 2016b, 2020; Sullivan et al., 2015). Interestingly, these studies suggest that temperamental differences can be detected already from the first year of life in infants with familial liability for ADHD (Miller et al., 2020). ASD and ADHD aggregate in families, which means that later-born siblings to a child with ASD are not only at elevated likelihood for the same disorder, but also for ADHD (Ghirardi et al., 2018; Miller, Degnan, et al., 2018; Miller, Musser, et al., 2018). Thus, infant sibling samples of ASD contain a significantly higher number of children with a higher likelihood for ADHD (either clinically or sub-clinically) than in the general population making this population suitable for studying both ASD and ADHD symptoms (Ali et al., 2020; Charman & Jones, 2019; Messinger et al., 2013; Miller et al., 2016a, 2016b; Piccardi et al., 2021). Understanding early risk factors for ADHD behaviors in infants at elevated likelihood for ASD can be of great importance not only theoretically, but also for early individually-tailored intervention.

Temperament is commonly defined as biologically-based reactivity and regulation of emotions, motor activity and attention (Rothbart & Derryberry, 1998). According to Rothbart’s influential model, three broad factors underlie temperament from infancy through late childhood (Rothbart, 1981; Rothbart & Bates, 2006). The first factor, *surgency*, concerns the child’s processing of potential reward, which triggers approach tendencies, positive affect and elevated activity level. The second factor, *negative affectivity*, concerns the child’s tendency to experience negative emotions, such as distress, sadness, fear, frustration and soothability. The third factor, *effortful* *control*, involves the child’s ability to regulate behavior, stay focused and control impulses. The two first reactivity factors (surgency and negative affectivity) can be identified during the first year of life whereas the third regulatory factor (effortful control), develops during the second year of life when the child becomes more consciously aware (Gartstein et al., 2013).

Many young children with ASD and ADHD share certain temperamental characteristics, such as high negative affectivity and low effortful control (Johnson et al., 2015). These temperament traits have been suggested to constitute transdiagnostic risk towards psychopathology in general (Hankin et al., 2017). Importantly, however, there seem to be differences in the behaviors underlying apparent similarities in these broad temperament factors in ASD and ADHD (Visser et al., 2016). For example, children with ASD often show negative affect through high levels of fear, distress and sadness (Garon et al., 2016; Jones et al., 2014), whereas high negative affect in children with ADHD are more related to irritability and anger (Karalunas et al., 2014; Martel & Nigg, 2006; Sullivan et al., 2015). Similarly, different aspects of effortful control have been found to relate to ASD and ADHD, with low social motivation and disengagement primarily relating to ASD, whereas difficulties with sustained attention and distractibility relate to children with ADHD (Visser et al., 2016).

Interestingly, a quite different pattern appears for surgency in relation to ASD and ADHD, both at the factor and behavioral level. There are some mixed findings in relation to ASD, with some studies indicating that infants who later develop ASD show more approach behaviors and higher activity level during the first year of life (Del Rosario et al., 2014; Garon et al., 2009; Zwaigenbaum et al., 2005). However, higher surgency in infants at elevated likelihood for ASD seem to be reversed during the second year of life with low levels of positive affect, approach and smiling (Del Rosario et al., 2014; Garon et al., 2009; Zwaigenbaum et al., 2005). In contrast, ADHD is often associated with high surgency as defined by elevated extraversion, activity level, approach and positive affect (Auerbach et al., 2008; Miller et al., 2016a, 2016b; Miller, Degnan, et al., 2018; Miller, Musser, et al., 2018).

Research examining temperament in children with ASD and ADHD has generally relied on indirect measures, particularly parent questionnaires (for a review see Kostyrka‐Allchorne et al., 2019). However, a few studies have used observational methods to explore early temperament in relation to ASD and ADHD (e.g., Hirschler-Guttenberg et al., 2015; Macari et al., 2018; Miller et al., 2020), although mostly focusing on negative behaviors, such as anger and frustration (Miller et al., 2020). While information from parents have been found to be useful, adding other measures could provide a more comprehensive evaluation of children’s temperament (Karp et al., 2004). In addition to parent-ratings of temperament we therefore measured some relevant temperament-related constructs via other means: direct observation and via an accelerometer. The observational measures were focused on the constructs of exuberance and activity level. Exuberance is closely related to Rothbart’s definition of surgency and is defined as extreme, uninhibited positive reactivity and strong approach tendencies (Polak‐Toste & Gunnar, 2006). Exuberant temperament is a relatively stable, multifaceted construct (Fox et al., 2001), driven by heightened sensitivity to reward (Polak‐Toste & Gunnar, 2006). Recent research has highlighted the importance and complexity of this construct as it has been found to be a potentiating factor for risk in certain contexts, and a resilience factor in others (Cicchetti, 2015). For example, positive emotionality in children promote later effortful control and is a cornerstone in developing positive peer relations (Gartstein et al., 2012). High levels of exuberance, on the other hand, is often expressed in situations when inappropriate and has been related to externalizing behaviors (Rydell, et al., 2003), low self-control and ADHD (Brocki et al., 2019).

Activity level is a component of both surgency and exuberance, isomorphic to one of the core dimensions of ADHD, and is suggested to be a reliable and stable early marker of later ADHD symptoms (Frick et al., 2018; Kostyrka-Allchorne et al., 2019). Moreover, increased activity level specifically predicted later ADHD symptoms in the context of infants at elevated likelihood for ASD (Shephard et al., 2018). However, these studies have generally relied on parent reports and we were therefore interested in examining the link between temperament and later symptom levels by direct observations and accelerometers, which has been used to assess movement patterns in various contexts (Yang & Hsu, 2010).

Against this background, the aim of the current study was to examine specific and common associations between early temperamental measures and symptoms of ASD and ADHD in a sample at elevated liklihood for ASD. We hypothesized that most of the parent-rated temperament behaviors included in Rothbart’s three factor model would specifically relate to either symptoms of ASD or ADHD symptoms when accounting for symptom overlap in these relations (please see Table 1 for our specific predictions for each of the potential temperament-symptom associations). We further predicted that exuberance and activity level would be related to higher ADHD symptoms, whereas potential relations between these measures and ASD symptoms formed an open question based on the inconsistencies in the literature.

**Table 1:** Hypotheses of the assumed directions between aspects of temperament at 18 months and ASD and ADHD symptoms at 36 months

Temperament	ASD symptoms	ADHD symptoms	ASD and ADHD symptoms
Parent-rated behaviors			
Surgency			
Sociability	−	+	
Positive anticipation	−		
Impulsivity		+	
Negative affectivity			
Fear	+		
Sadness	+		
Frustration		+	
Anger		+	
Effortful control			
Attention shifting	−		
Inhibitory control	−	−	−
Cuddliness		−	
Attentional focus	−	−	−
Observed behaviors			
Exuberance			
Exuberance		+	
Activity level		+	
Objective activity level			
Activity level			
Accelerometer activity		+	

The minus sign (−) represents a negative association, whereas the plus sign (+) represents a positive association to later symptoms of ASD, ADHD specifically, and symptoms of ASD and ADHD combined

## Method

### Participants

The participants were part of an ongoing longitudinal project, the Early Autism Sweden (EASE), following infants from 5 months until 6 years of age. Here, we use data from assessments at 18- and 36-months of age. The reason for selecting the 18-months assessment was that we wanted to use the bubble task in the ADOS-2 as an early indicator of exuberance/activity, and 18 months is the earliest time point we have this measure on all participants. The 36-month (rather than the 6 year) time point was selected to increase sample size for the analysis (most children have not yet reached 6 years). Informed consent was given by the parents prior to conducting the assessments.

At time of enrollment, the infants had at least one older sibling diagnosed with ASD, based on the presence of a community clinical diagnosis. The majority of the community clinical assessments for the diagnosed sibling included both the ADOS-2 and ADI-R, considered gold standard measures for diagnosing ASD. Exclusion criteria at the point of recruitment included birth before 36 weeks of gestation, visual or auditory impairment, or any known medical or genetic conditions. Families in the study were recruited from clinical units, advertisements and the project’s web page. Socioeconomic status was calculated as the mean of level of family income rated on a scale from 0 to 5 and parent educational level rated on a scale from 1 to 5, expressed as Z scores and is reported in Table 2 together with developmental level, as measured by the Mullen Scales of Early Learning Composite score (MSEL; ELC; Mullen, 1995). Since we were interested in dimensional associations with both ASD and ADHD we used continuous measures of symptoms (and not clinical diagnosis).

**Table 2:** Participant characteristics (mean/SD) of infant siblings at 18 months and 36 months

	M/SD	Range	Skewness/SE	Kurtosis/SE
18-months				
Age at 18 months/days	556.57/16.95	506–607		
MSEL ELC 18 months	95.15/15.3	67–133	0.08/0.28	− 0.73/0.56
SES^2^	− 0.09/.87	1–5	− 0.95/0.27	− 0.33/0.54
36-months				
Age at 36 months/days	1113.43/30.75	1045–1253		
CBCL-ADHD scale^3,4^	3.49/2.87	2.60–4.38	0.75/0.31	− 0.52/0.61
CBCL-PDP scale ^3,4^	3.40/3.98	2.17–4.62	2.07/0.31	4.50/0.61
C-TRF ADHD scale ^3,5^	4.19/5.48	2.50–5.87	1.61/0.35	1.92/0.68
C-TRF PDP scale ^3,5^	3.14/3.94	1.93–4.35	1.22/0.35	0.92/0.68
CBCL/C-TRF ADHD^3,6^	3.56/3.96	2.56–4.55	1.71/0.30	3.40/0.60
CBCL/C-TRF PDP^3,7^	3.17/3.05	2.40–3.95	1.03/0.30	0.37/0.60

^1^Mullen scales of Early Learning composite score, ^2^Socioeconomic status was calculated as the mean of level of family income rated on a scale from 0 to 5 and parent educational level rated on a scale from 1 to 5, expressed as Z scores, ^3^Raw scores, ^4^Child Behavior Checklist for ages 1 1/2–5 years (rated by parents), ^5^Caregiver-Teacher Report Form for ages 1 1/2–5 years (rated by daycare providers and teachers), ^6^Joint measure of CBCL and C-TRF ADHD scales, ^7^Joint measure of CBCL and C-TRF PDP scales

*PDP*  Pervasive Developmental Problems

The initial sample consisted of 86 children at elevated likelihood for developing an ASD diagnosis. Out of these, 9 children were excluded from the analysis because of technical problems during video-recording resulting in a sample of 77 children. A drop-out analysis showed that there was no difference between the excluded and the included children who had data on the 18 months CBCL scales for ADHD (*t* = 0.40, *p* = 0.68) and Pervasive Developmental Problems (PDP; *t* = 1.37, *p* = 0.18). Out of the 74 children of the 77 children who had diagnostic data at 3 years, 27 (36%) children had received an ASD diagnosis at 3 years. The control group, consisting of children with typical likelihood, was too small (N = 22) to make proper comparisons, and hence not included in the analysis. In addition, our research questions motivated examining the group of children at elevated likelihood for ASD specifically. Descriptive and inferential statistics on background data is summarized in Table 2 and temperament data is summarized in Table 3.

**Table 3:** Descriptive statistics (mean/SD) of predictor variables at 18 months

	M/SD	Range	Skew	Kurtosis
Parent-rated temperament				
Inhibitory control	3.39/1.06	1.42–5.17	− 0.21	− 1.09
Attention shifting	4.55/0.90	2.40–6.43	− 0.55	0.12
Low-intensity pleasure	4.80/1.00	1.64–6.70	− 0.83	1.63
Cuddliness	5.25/0.75	3.50–6.67	− 0.46	− 0.40
Attentional focusing	3.54/0.99	1.00–5.42	− 0.67	0.17
Discomfort	1.96/0.70	1.00–3.83	1.13	0.92
Fear	2.04/0.70	1.00–3.86	0.72	0.14
Motor activation	1.58/0.48	1.00–3.33	1.47	2.47
Sadness	3.22/0.78	1.20–5.20	0.28	0.42
Perceptual sensitivity	2.86/1.27	1.00–6.00	0.71	− 0.40
Shyness	4.37/0.94	1.00–6.75	− 0.13	0.47
Soothability	5.70/0.83	3.40–7.00	− 1.20	1.46
Frustration	3.57/0.82	1.83–5.09	0.12	− 0.91
Impulsivity	4.94/0.97	2.14–6.56	− 0.67	0.28
Activity level	4.66/0.92	3.00–6.50	0.05	− 0.97
High-intensity pleasure	4.37/0.94	1.75–6.40	− 0.13	0.08
Sociability	4.88/1.32	1.33–7.00	− 0.88	0.44
Positive anticipation	4.32/1.44	1.00–7.00	− 0.62	0.09
Observed behaviors				
Exuberance	3.11/0.98	1.00–5.00	− 0.13	− 0.30
Activity level	2.98/0.99	1.00–5.00	0.08	− 0.25
Accelerometer activity	9.99/3.04	4.25–19.15	0.60	0.58

Ethical approval was given by the Regional Ethics Board in Stockholm, Sweden (Dnr. 2010/2085-31/3). The study was conducted in accordance with the 1964 Declaration of Helsinki.

## Predictors at 18-Months

### Observed Behaviors of Temperamental Exuberance

Temperamental exuberance was assessed from the bubbles task in the ADOS-2 Toddler (Lord, Rutter, DiLavore, Risi, Gotham, & Bishop, 2012). We chose this task due to the similarities with the bubble task used to measure exuberance in the Laboratory Temperament Assessment Battery (Lab-Tab; Goldsmith & Rothbart, 1996), a standardized task with a valid coding schedule. We used the coding schedule from the Lab-Tab as a template for developing a customized coding schedule that was better suited for the bubble task in ADOS-2 which we used in the current study. In this task an examiner blows bubbles and plays with the child for a brief period of time. The task also involves a part where the examiner “teases” the child by first offering the bubble machine to the child and then removing the bubble-toy when/if the child reaches for the toy. A customized coding schedule was developed in order to catch relevant behaviors from the bubble task in ADOS-2 (see Supplementary Material for the coding criteria and protocol). Activity level, positive affect and sociability were coded in 15-s intervals on a 1–5 scale, with 1 being the lowest score and 5 being the highest score. To use activity level as an example, we used the following criteria; (1) represents no motor activity (e.g., child is completely or almost completely motionless, does not manipulate or actively play with toys), (2) describes limited motor activity (e.g., child walks around the room or plays with low intensity), (3) was used for moderate motor activity (e.g., continuous and relaxed motor activity), (4) was used for active motor activity (e.g., child walks quickly, jumps, or show occasional high intensity movements), and (5) was used for intense motor activity (e.g., child rushes around or plays with exaggerated intensity/energy). See Supplementary Material for full descriptions of the scales. The segment codes were then used to decide on a global score for each variable, which in turn were used as dependent measures. The rationale for this approach was that some children may take time to warm up, wherefore an estimate global score seemed more appropriate to use than computing averages based on the segments. The global score was a total score based on the total 2.5-min task for each variable coded on the same 1–5 scale as for the segment scores. We used the global score as a dependent measure for this task.

The scales Bodily fear, Facial sadness and Distress were omitted from further analyses due to no variations in this particular task. The scales Proximity to parent, Latency to approach and Degree of approach was omitted from further analyses to limit the amount of analyses. Three coders, unaware of ASD likelihood status, were initially trained to reach acceptable levels of agreement. Double-coding was done on 43% (*N* = 30) of the data. Intraclass correlation (ICC) estimates were calculated based on the mean-rating (*k* = *3*) absolute agreement, 2-way mixed effect model. The average ICC coefficients were 0.76 for activity level, 0.88 for positive affect, and 0.70 for sociability. All ICCs were considered as indications of good interrater reliability (Cicchetti, 1994). The global coding of the 2.5 min sequence for Activity level, Positive affect and Sociability were highly correlated (*r* = 0.67–0.78) and a mean estimate was used as a score of exuberance.

### Parent-Rated Behaviors Underlying Rothbart’s Three Factor Model of Temperament

The Early Childhood Behavior Questionnaire (ECBQ; Putnam et al, 2006) is a reliable and well-validated parent-report questionnaire developed to target temperament according to Rothbart’s three factor model in toddlers between 18 and 36 months of age. The ECBQ contains 18 dimensions based on 201 items (the dimensions are outlined in supplementary material). Parents rate their infants’ behavior over the past 2 weeks on a 1–7 scale reflecting frequency. We used the underlying behaviors as predictors at 18 months in the present study. Cronbach’s alpha for the behaviors included in the Surgency factor were; α** = **0.71 for Impulsivity, α** = **0.76 for Activity level, α** = **0.91 for High-intensity pleasure, α** = **0.96 for Sociability, and, α** = **0.81 for Positive anticipation. Cronbach’s alpha for the behaviors included in the Negative affectivity factor were; α** = **0.63 for Discomfort (after removing one item which decreased alpha to unacceptable level), α** = **0.65 for Frustration, α** = **0.72 for Motor activation, α** = **0.81 for Perceptual sensitivity, α** = **0.88 for Shyness, and α** = **0.88 for Soothability. Finally, Cronbach’s alpha for the behaviors included in the Effortful control factor were; α = 0.92 for Inhibitory control, α = 0.86 for Attention focusing, α = 0.75 for Attention shifting, α = 0.86 for Low-intensity pleasure, and α = 0.90 for Cuddliness. Alpha levels for all scales fell within the reasonable to excellent reliability range (Taber, 2017).

### Physiological Activity Level

To estimate the children’s overall movement activity level during the whole visit (i.e. around 3–5 h), we used small portable accelerometers (Q-Sensors, Affectiva, Inc.; Waltham, MA) which recorded the 3D acceleration of the arm. The Q-sensors are worn on the wrist of the child, and records arm movements by accelerometers along the x, y and z axis of the device in gravity units (i.e. a completely still sensor records 1 G). Movement activity level was calculated by transforming these axes to a three-dimensional space and computing the root mean square (RMS) of all samples in the 3D acceleration profile. Because some of the infants took a nap during the visit, the acceleration time series were segmented into bins with a duration of 1 min each, and completely passive bins (RMS < 0.05, after removing the average of the raw 3D acceleration) were excluded from the RMS calculations. These thresholds were determined based on visual inspection, blinded for risk group and outcome data. The data was recoded with a sample rate of 32 Hz.

### Developmental Level

Mullen Scales of Early Learning (MSEL; Mullen, 1995) is a standardized measure of development for children from birth to 68 months of age in the domains of fine and gross motor skills, visual reception, expressive and receptive language. The Early Learning Composite (ELC) is a standard score (M = 100, SD = 15) for overall cognitive ability, which was used in the current study for participant characteristics.

## Continuous Outcome Measures at 36-Months

### Parent- and Teacher-Ratings of ASD and ADHD Symptoms

The Child Behavior Checklist (CBCL) 1½–5 and the Caregiver-Teacher Report Form (C-TRF) are similarly constructed to evaluate behavioral, emotional and social function problems (Achenbach & Rescorla, 2000). Both scales have good reliability and validity (Achenbach 1991b). In the current study, the CBCL was rated by parents and the C-TRF was rated by teachers and equivalent pre-school staff. Both forms comprise 99 items that are rated on 0 (not true), 1 (somewhat or sometimes true), or 2 (very or often true). In the present study we used the DSM-related scales of Pervasive Developmental Problems (PDP) as a proxy for ASD symptoms, and the Attention deficit/Hyperactivity problem scale as a proxy for ADHD symptoms. The PDP scale is identical to the DSM ASD-scale in the recent CBCL 1½–5 C-TRF version (Achenbach, 2014) apart for the item “afraid of trying new things” that is not included in the recent version. The ADHD scale is identical in both versions. In this study we used a multiple-informant approach by aggregating continuous outcome measures of ASD and ADHD symptoms based on parent- and teacher-ratings. Although disaggregated data may produce more specific report from different informants, in this study we chose to aggregate data in order to present a comprehensive measure of behaviors across setting and to reduce the number of analyses and the risk of type I errors (Holmbeck et al., 2002). In line with studies using a multi-informant approach (e.g., Achenbach et al., 1987; Schniering et al., 2000) we reasoned that incorporating both parents and teachers would increase the comprehensiveness of the data by providing multiple perspectives regarding the same behavior. The parent-ratings (CBCL) and the teacher-ratings (C-TRF) of the ADHD and PDP-scales were moderately correlated (*r* = 0.55, *p* = 0.001 for the ADHD-scales; *r* = 0.43, *p* = 0.008 for the PDP-scales) and in all of the following analyses a composite score for the symptom scales calculated as a mean score of the symptom scales of CBCL (rated by parents) and C-TRF (rated by teachers) was used.

### Statistical Analyses

Data were converted to z-scores and screened for outliers (z > 3 SD). Six extreme univariate outliers were detected, one on the temperamental scale attentional shifting, one on the cuddliness scale, one on the discomfort scale, one on the soothability scale, and two on the CBCL scale Pervasive Developmental Problems (ASD symptoms). These outliers were replaced with the value next most extreme for each measure (Tabachnick & Fidell, 2013). Analysis for normality showed that the collapsed symptom scales of ASD and ADHD (representing averaged parent- and teacher-ratings from the CBCL and C-TRF) and the parent-rated temperament scales of discomfort, frustration, low-intensity pleasure, repetitive motor activity/fidgeting, sociability and soothability were positively skewed. To correct for this non-normal distribution bootstrapped statistical tests were used in the analysis involving these variables (see Field, 2013). Interrelations between the predictors at 18 months and longitudinal relations between the predictors and ASD and ADHD symptoms were analyzed using bootstrapped Pearson’s correlations. To test the unique relations between the predictor variables and each of the outcome variables (ADHD vs ASD traits), bootstrapped partial correlations were used with control for ASD in the relations to ADHD and vice versa. To adjust for multiple testing for the temperament dimensions in the *longitudinal* correlation analyses we used the more conservative alpha of 0.01 as significance level (exact p-values are reported). This alpha-level was considered a reasonable correction without excessively increasing the risk of inflated type II errors. If participants have skipped an item on any of the rating-scales, they will be prompted to fill in that/those items before finishing the questionnaire. This reduces the amount of missing data. However, due to failure of parents and/or teachers to submit ratings on the ECBQ and/or CBCL, 14 children had missing data on these variables, however, for the temperament scales impulsivity, positive anticipation, and sociability 16, 18 and 15 children had missing data, respectively. Due to internal omission, 28 children had missing data on the exuberance measure from the bubble-task, and 23 children had missing data on the accelerometer activity measure. Therefore, total *n* for the bivariate analyses varied between 42 and 54.

All procedures performed in studies involving human participants were in accordance with the ethical standards of the institutional and/or national research committee and with the 1964 Helsinki declaration and its later amendments or comparable ethical standards.

## Results

Descriptive statistics for participant characteristics are presented in Table 2. Regarding correlations at 18 months, we found that increased accelerometer activity level was related to increased observed activity level, *r*(41) = 0.34, *p* = 0.04, parent-rated activity level, *r*(41) = 0.34, *p* = 0.02, indicating construct validity in the rated activity variables. Increased accelerometer activity level was also related to decreased parent-rated attentional focus, *r*(42) = 0.32, *p* = 0.03. Exuberant children in the bubble-task showed less fear as rated by the parent, *r*(42) = 0.29, *p* = 0.04. No other correlations were significant between the predictors (observations, ratings and accelerometer activity level) at 18 months. At 36 months, symptoms of ASD and ADHD were positively correlated, *r*(51) = 0.55, *p* < 001.

### Longitudinal Relations Between Temperament at 18 Months and Symptoms of ASD and ADHD at 36 Months

Longitudinal relations between the multi-measured temperament predictors at 18 months and ASD and ADHD symptoms at 36 months are presented in Table 4. As for the separate behaviors making up the effortful control factor, we found that increased inhibitory control was related to decreased ADHD symptoms, *r*(52) = − 0.49, *p* = 0.001, and this relation remained when partialling out the effect of ASD symptoms, *r*(51) = − 0.49, *p* = 0.001. Increased attentional shifting was related to decreased symptoms of ASD, *r*(52) = − 0.39, *p* = 0.001 and survived control for symptom overlap, *r*(51) = − 0.31, *p* = 0.01.

**Table 4:** Bootstrapped correlations between rated and observed temperament, exuberance and activity level at 18 months and ASD and ADHD symptoms at 36 months for the infant siblings

Predictor variables	ASD Symptoms		ADHD Symptoms	
	*r*	*p*	*r*	*p*
Parent-rated effortful control				
Inhibitory control	− 0.13 (0.14)	0.33 (0.30)	− **0.49 (**− **0.49)**	**0.001 (0.001)**
Attention shifting	− **0.39 (**− **0.31)**	**0.001 (0.01)**	− 0.28 (− 0.11)	0.02 (0.32)
Low-intensity pleasure	− 0.29 (− 0.18)	0.03 (0.21)	− 0.29 **(**− 0.18)	0.03 (0.20)
Cuddliness	− 0.29 (− 0.22)	0.03 (0.12)	− 0.16 (− 0.05)	0.02 (0.55)
Attentional focus	− 0.10 (− 0.01)	0.47 (0.97)	− 0.19(− 0.17)	0.16 (0.24)
Parent-rated negative affectivity				
Discomfort	**0.42** **(0.43)**	**0.002 (0.001)**	0.13 (− 0.06)	0.49 (0.30)
Fear^a^	0.19 **(0.33)**	0.17 **(0.01)**	− 0.19 **(**− 0**.33)**	0.17 **(0.01)**
Motor activation	− 0.09 (− 0.01)	0.52 (0.94)	0.16 (0.14)	0.24(0.33)
Sadness	0.07 (0.04)	0.62 (0.78)	0.07 (0.04)	0.62 (0.78)
Perceptual sensitivity	− 0.02 (0.15)	0.90 (0.27)	− 0.23 (− 0.27)	0.10 (0.05)
Shyness	0.21 (0.29)	0.13 (0.04)	− 0.08 (− 0.21)	0.58 (0.13)
Soothability	− **0.33** (− .19)	**0.01** (0.20)	− **0.36 **(− 0.24)	**0.008 **(0.08)
Frustration	0.22 (0.06)	0.11 (0.64)	**0.34 **(0.27)	**0.01 **(0.05)
Parent-rated surgency				
Impulsivity	− 0.22 **(**− **0.35)**	0.12 **(0.01)**	0.14 (0.32)	0.32 (0.03)
Activity level	**0.38 **(0.14)	**0.007 **(0.35)	**0.53 (0.42)**	**0.001 (0.003)**
High-intensity pleasure	0.19 (− 0.03)	0.18 (0.81)	0.31 (0.25)	0.03 (0.08)
Sociability	− **0.47 (**− **0.43)**	**0.001** **(0.002)**	− 0.21 (0.04)	0.13 (0.76)
Positive anticipation	0.02 (− 0.01)	0.91 (0.96)	0.02 (0.01)	0.89 (0.93)
Observed behaviors				
Exuberance	0.17 (0.06)	0.08 (0.70)	0.31 (0.27)	00.05 (0.09)
Activity level	0.34 (0.26)	0.03 (0.09)	0.27 (0.16)	0.06 (0.24)
Objective activity level				
Accelerometer activity	0.29 (0.05)	0.06 (0.77)	**0.38 **(0.26)	**0.01 **(0.10)

Figures within parentheses represent bootstrapped partial correlations adjusted for co-occurring ASD symptoms and ADHD symptoms, respectively

To adjust for multiple comparisons, the results are interpreted as significant if p <0 .01 as marked in bold.

^a^Pearson’s *r* and *p*-values for fear are, although strikingly similar for ASD and ADHD, correct

In terms of the behaviors constituting the negative affectivity factor, increased discomfort was related to increased symptoms of ASD, *r*(52) = 0.42, *p* = 0.002, and this relation survived control for ADHD symptoms, *r*(51) = 0.43, *p* = 0.001. Increased infant soothability related to decreased ASD, *r*(52) = − 0.33, *p* = 0.01, and ADHD symptoms, *r*(52) = − 0.36, *p* = 0.008, but these relations did not hold after controlling for the overlap of respective symptom domain. The bivariate relations between fear and symptoms of ASD and ADHD were not significant, but after control for symptom overlap the relations became significant albeit in the opposite direction for the two symptom domains, that is increased fear was related to increased ASD symptoms, *r*(52) = 0.33, *p* = 0.01, and decreased ADHD symptoms, *r*(52) = − 0.33, *p* = 0.01.

Several behaviors belonging to the surgency factor were related to both ASD and ADHD symptoms. First, the bivariate relations between impulsivity and ASD and ADHD symptoms were not significant, but when controlling for symptom overlap, the relation between increased impulsivity and decreased ASD symptoms became significant, *r*(48) = − 0.35, *p* = 0.01. Further, increased parent-rated activity level was related to increased ASD, *r*(49) = 0.38, *p* = 0.007, and ADHD symptoms, *r*(49) = 0.53, *p* = 0.001, but only the relation to ADHD survived control for symptom overlap, *r*(48) = 0.42, *p* = 0.003. Finally, increased sociability was related to decreased ASD symptoms, *r*(49) = − 0.47, *p* = 0.001, and this relation remained when controlling for ADHD symptoms, *r*(48) = − 0.43, *p* = 0.002.

In terms of observed exuberant behaviors in the bubble task, neither exuberance nor observed activity level were significantly related to ASD or ADHD behaviors. Activity level as measured by the accelerometer was related to increased ADHD symptoms, *r*(42) = 0.38, *p* = 0.01, but this relation disappeared when controlling for ASD symptoms.

## Discussion

It is still unclear whether ASD and ADHD characterize different manifestations of the same overarching disorder or if co-occurring ASD and ADHD represent different clinical disorders with distinct etiology and developmental trajectory (Rommelse et al., 2011; Leitner, 2014). In this study, the overall pattern of results indicate that the specific behaviors underlying Rothbart’s three temperament factors—effortful control, negative affectivity and surgency, were uniquely related to *either* later ASD or ADHD symptoms. These findings suggest that particular early temperamental behaviors may differentiate between early emerging ASD and ADHD symptoms.

The pattern of results in the present study point to specific temperamental features of ASD and ADHD symptoms and add to the current literature on specific developmental pathways to these conditions (Johnson et al., 2015; Shephard et al., 2018; Visser et al., 2016). Recent research with a focus on the broad temperament factors have revealed similarities in low effortful control and high negative affectivity in relation to ASD and ADHD, but our results suggests that these apparent similarities might be driven, at least in part, by different underlying behaviors. For example, a reduced ability to regulate (i.e., low effortful control) is a consistent finding in the literature of both ASD and ADHD (Jahromi, 2017; Nigg, 2006; Nigg et al., 2004). In contrast, our findings, based on the behavioral subscales rather than on Rothbart’s overarching factors, suggest that different regulatory aspects are related to ASD vs ADHD. More precisely, poor inhibitory control was clearly and specifically related to ADHD symptoms, which is a consistent finding in the ADHD literature (Barkley, 1997; Brocki et al., 2007), whereas poor attention shifting was specifically related to ASD symptoms, a finding in line with recent empirical work (Hendry et al., 2020; Johnson et al., 2015). We found no association between attentional focusing and ASD or ADHD symptoms, a finding which is in line with Shephard et al., (2018) who reported null-associations between the same ECBQ measure at 24 months and diagnosis of ASD or ADHD at age 7 in a sample including children at an elevated likelihood for later diagnosis. Together, these findings suggest that although theoretically relevant for both disorders, the temperamental aspect attentional focusing in toddlerhood as measured by parent-ratings at one time point is not a sensitive measure for predicting later ASD or ADHD.

For negative affectivity, our results point to both similarities and differences in relation to symptoms of ASD and ADHD. Poor soothability was related to both ASD and ADHD symptoms before controlling for the overlap of respective symptom domain. This result aligns with past studies suggesting that soothing difficulties is a more general and transdiagnostic trait related to many psychopathological conditions (Visser et al., 2016). However, discomfort, defined as the expression of negative affect in terms of sensory qualities of stimulation such as light, sound and/or texture (Rothbart, 1981) was specifically associated with ASD symptoms and not ADHD symptoms. Elevated levels of discomfort tap into the concept of hypersensitivity which is part of the diagnostic criteria for ASD (Tomchek, & Dunn, 2007), whereas negative emotionality and irritability are common characteristics of the ADHD phenotype (Karalunas et al., 2014; Martel & Nigg, 2006). However, we were unable to find a specific link between frustration and ADHD symptoms. Fear and anger are two suggested pathways that lead to internalizing and externalizing disorders (including ADHD) respectively (Auerbach et al., 2008; Rothbart & Bates, 2006). Interestingly, we found that fear was specifically related to symptoms of both ASD and ADHD, although in the opposite direction. That is, when controlling for the overlap between the two symptom domains, fear related to increased ASD symptoms and decreased ADHD symptoms. The heightened levels of fear in relation to ASD symptoms confirmed our prediction as well as earlier findings of higher fear levels in relation to ASD (Garon et al., 2016; Jones et al., 2014). Due to the limited amount of research on fear and ADHD in younger children we did not form a prediction regarding this potential relation. However, the negative association between fear and later ADHD symptoms in our sample may well be an early aspect of the fearlessness and risk-taking behavior that is often characteristic of individuals with ADHD in childhood and adulthood (Pollak et al., 2019). This finding is particularly important as it points to strong and early specificity for the different ends of the fear construct, high vs low levels, in relation to later development of ASD and ADHD symptoms, respectively, and as such may be a potential early and specific risk marker. Again, this pattern of results suggest that there are different behaviors that drive the effect of the broad temperament factors negative affectivity, surgency and effortful control, emphasizing that these behaviors need to be thoroughly defined both in respect to theory and intervention development.

Although only a limited amount of research has explored surgency and exuberant behaviors in relation to ASD and ADHD, the extant studies on this topic point to differences in the directions of these relations. Low surgency, such as low rates of smiling and positive expressions have been associated with ASD (Garon et al., 2009, 2016) and high rates of surgency, such as exuberant behaviors and difficulty regulating positive emotions, have been related to ADHD (e.g., Brocki et al., 2019; Forslund et al., 2016). Our results, showing low levels of impulsivity and sociability being specifically linked to ASD symptoms and activity level being specifically linked to ADHD symptoms, indicate that there are certain differences in surgent behaviors at 18 months which distinguish between ASD and ADHD symptoms in children at 3 years. Reduced sociability, and specifically deficits in social communication and social interaction, are diagnostic symptoms of the ASD criteria (DSM V, 2013) and consistent with past work on early temperament in infants at elevated likelihood for ASD (e.g., Paterson et al., 2019; Visser et al., 2016). Interestingly, while impulsivity is closely linked to the diagnostic criteria for an ADHD diagnosis (DSM-5, 2013), it has not been thoroughly investigated as a potential early marker in relation to ASD. However, given that the definition of impulsivity relates to speed of response initiation, this finding might partially overlap with the impaired initiation processes found in ASD (Bramham et al., 2017).

Moreover, observed exuberance did not meet our conservative 0.01 criteria for significance. Thus, we were not able to find support for the hypothesis that exuberance could be an alternative pathway to ADHD symptoms, at least not in our sample of young siblings at elevated likelihood for ASD. There are several potential reasons for this result, it may depend on our modest sample size or that the observation measure did not capture the full range of exuberant behavior in toddlers. Parents rate their children based on a broad range of different situations while our observation measure reflects the childrens’ behavior in a very specific situation in a new context which may not be representative for their behaviors in general. Lastly, the children in our sample were recruited based on their older sibling with a diagnosis of ASD, and the result might be different in a familial risk study of siblings with ADHD.

Parent-rated activity level was uniquely related to ADHD symptoms after control for ASD symptom overlap. We also found a bivariate association between accelerometer activity level and ADHD symptoms, which did not survive control for ASD symptoms. The observed activity level in the bubble task was not related to later ADHD symptom level. These findings suggest that parental insights into their child’s level of activity may offer a possibility to identify this early behavioral marker. Indeed, parent-ratings are time-effective and implemented at a low-cost, which is beneficial in research as well as in the clinic. The association between activity level and ADHD symptoms a well-established finding in the ADHD literature and part of the diagnostic criteria (DSM-5, 2013). Activity level is suggested to predict later ADHD symptoms already from infancy to school-age, and up to adolescence both dimensionally and categorically (Frick et al., 2018; Kostyrka-Allchorne et al., 2019; Auerbach et al., 2008; Sonuga-Barke, 2005, Einziger et al., 2018).

It should be mentioned that our results are not uniform and point to slight inconsistencies across the different methods. There are various potential explanations to these inconsistencies. First, and as mentioned previously, parents evaluate their child’s behavior in varied contexts and situations during longer time-intervals whereas examiner-ratings during lab-visits capture the child’s behavior under limited circumstances. These variations in methods may capture behaviors related to trait (part of a long-term characteristic of an individual) vs state (a temporary condition) qualities in the child. The observational measure was primarily used to assess one aspect of temperament, namely exuberance. Indeed, it might be that other types of tasks could elicit more exuberant behaviors and a future direction would be to use a variety of measures to assess this temperamental trait. Moreover, we studied physical activity using a triaxial accelerometer for movement registration. Although this method has been used to assess movement patterns in various contexts (Yang & Hsu, 2010), it has not been commonly used in developmental psychopathology research. The accelerometer data provides a rough measure of activity level of the child since it was measured throughout a full testing-day at the lab. Indeed, we were able to find relations between all activity level measures (parent-ratings, observations and accelerometers) which indicate some construct convergence between these measures. Although we found a relation between accelerometer activity level and later ADHD symptoms, the association did not hold when controlling for overlapping ASD symptoms. In the current study we were unable to perform more fine-grained analyses of the accelerometer data, to assess the variability pattern during a testing day and to split the accelerometer data by task. Clearly, this limitation may obscure important information in the data, and should be addressed when planning future studies.

It is worth noting that some of the temperament constructs overlaps with the diagnostic criteria of ADHD, specifically impulsivity, attentional focusing and activity level. Indeed, we found a strong relation between parent-rated activity level at 18 months and later ADHD symptoms, but only marginal relations between attentional focusing and impulsivity at 18 months in relation to later ADHD symptoms. Indeed, ADHD has been characterized as the extreme end of child temperament (Foley et al., 2008) and following this line of reasoning our result could suggest that temperament, or symptom-related traits of ADHD can be spotted as early as 18 months of age.

In sum, while most studies on toddler temperament have relied on single measures such as parent-ratings, our study adds to the literature by using multiple measures such as observations and accelerometers in addition to parent-ratings. Indeed, parent-ratings provided the strongest associations between temperament at 18 months and symptoms at 36 months. Importantly, the associations between parent-rated behavioral features in toddlers at elevated likelihood for ASD and ADHD symptoms suggests that interventions could be individually tailored based on specific early behaviors in order to reduce symptom expression. This information may be particularly useful when it comes to providing early interventions.

### Limitations, and Future Directions

The goal of this study was to study links between temperament and ASD vs ADHD symptoms in infant siblings of children with ASD, i.e. primarily at elevated likelihood for ASD. Thus, the observed patterns of results need to be understood in this context. This being said, the overlap between the disorders, both in etiology and clinical expression (co-morbidity) are extensive, and thus it is not unlikely that some of the results generalize to other groups (e.g. primarily at elevated likelihood for ADHD) or to the population more broadly. This is an important avenue for future research. Another point of concern is that our sample is slightly skewed towards high socioeconomic status (SES) which suggests that the families in our study generally experience less economic burden in contrast to low SES families. There is an association between socioeconomic disadvantage and higher prevalence of ADHD (Russell et al., 2016) and ASD (Rai et al., 2012) and our results need to be considered in relation to this context.

Although temperament is supposed to emerge early in infancy before core symptoms of ASD and ADHD is measurable, our study design does not allow for a strong test of causal direction. Therefore, the links between temperament at 18-months and symptoms at 36-months are best understood as associations rather than cause- and effect- relations.

The multiple-measure approach gives an advantage over the typical study approach of using single measures. Here, we aimed at exploring the full range of parent-rated temperament behaviors that constitutes the three broad factors wherefore we ended up with multiple comparisons in relation to the sample size. In order to address the problem with multiple comparisons, we used a more conservative alpha level (i.e., 0.01). Some of the expected associations, such as the observed exuberance and activity level, were not significant at this threshold and did not clearly show a specific pattern to either ASD or ADHD-symptomatology. More well-powered studies need to further disentangle this question. By using a multi-informant approach (combined parent- and teacher-ratings) we were able to include two perspectives of the symptom level, however a way forward would be to also include clinical estimates of symptoms and finally diagnostic outcome.
